# Pharmacokinetic Exposures Associated With Oral Administration of Sorafenib in Dogs With Spontaneous Tumors

**DOI:** 10.3389/fvets.2022.888483

**Published:** 2022-05-19

**Authors:** Jacob R. Cawley, Samuel D. Stewart, Jonathan Paul Mochel, Sridhar Veluvolu, Chand Khanna, Joelle M. Fenger

**Affiliations:** ^1^Ethos Veterinary Health, Woburn, MA, United States; ^2^Ethos Discovery (501c3), San Diego, CA, United States; ^3^SMART Pharmacology, Department of Biomedical Sciences, College of Veterinary Medicine, Iowa State University, Ames, IA, United States; ^4^William R. Pritchard Veterinary Medical Teaching Hospital, School of Veterinary Medicine, University of California, Davis, Davis, CA, United States

**Keywords:** sorafenib, multi-target kinase inhibitors, pharmacokinetics, dog, cancer

## Abstract

Sorafenib is a multi-kinase small molecule inhibitor that targets serine/threonine and tyrosine kinases including the RAF kinase family, VEGFR-2, and PDGFR. The aim of this study was to evaluate the systemic pharmacokinetics of a previously defined tolerable oral dose of sorafenib in tumor-bearing dogs. Six client-owned dogs with a cytologic or histologic diagnosis of cancer were enrolled in this open-label, tolerability study. Dogs were administered sorafenib at an intended dose of 3 mg/kg and serum samples were obtained for analysis of sorafenib serum concentrations at 0, 1, 2, 6, 12, 24, 48, 72, 96, and 168 h post-drug administration. Median time to peak serum sorafenib concentration occurred at 4 h (range 2–12 h) resulting in an average serum concentration of 54.9 ± 33.5 ng/mL (118.2 ± 72.1 nM). Mean sorafenib levels declined by over 70% relative to peak serum concentrations by 24 h in all dogs, suggesting the value of at least twice daily administration. Doses of 3 mg/kg were well-tolerated and no patients in the study experienced adverse events that were attributable to sorafenib. Future trials in dogs with cancer are recommended at this dosing schedule to assess the effect of sorafenib administration on anti-tumor efficacy signals and relevant pharmacodynamic target modulation *in vivo*.

## Introduction

Sorafenib is an oral multi-kinase small molecule inhibitor that targets several serine/threonine and tyrosine kinases involved in tumor proliferation and angiogenesis, including Raf, VEGFR-2, VEGFR-3, and PDGFR-B. Sorafenib is a potent inhibitor of several Raf kinase isoforms, including C-Raf, wild-type B-Raf and mutant B-Raf (e.g., V600E mutant *b-raf*), as evidenced by reduced phosphorylated (p)ERK levels and proapoptotic effects in human colon and pancreatic tumor cells (both K-*ras*), melanoma cells (*b-raf* V600E), and breast cancer cell lines (K-*ras* and *b-raf* G463V) at low nanomolar concentrations *in vitro* (1, 2). In tumor xenografts, sorafenib has demonstrated preclinical activity in several advanced solid tumor models harboring oncogenic *b-raf* or K-*ras* mutations or increased signaling through Raf due to upstream receptor tyrosine kinase overexpression (3–5). In human cancer patients, sorafenib is approved by the U.S. Food and Drug Administration (FDA) for the treatment of advanced hepatocellular, thyroid, and renal cell carcinomas.

Preliminary studies with oral administration of sorafenib in rodents and dogs have demonstrated acceptable tolerability in both species. In human patients, the most common adverse events associated with sorafenib include hand-foot skin reaction (HFSR), diarrhea, hypertension, fatigue, abdominal pain, and nausea (6). Previous preclinical toxicity studies conducted in purpose-bred research dogs found that sorafenib administered orally at dosages of 30 mg/kg/day was associated with significant gastrointestinal, cutaneous, renal, and hematologic toxicity following long term administration (3–12 months); however, dose reductions below 10 mg/kg/day (a decrease of 67% of the original dose) were well-tolerated with only mild side effects including diarrhea, reduced body weight, and emesis (5). To date, sorafenib has been evaluated clinically in early phase tolerability studies in pet dogs with cancer. Administration of single doses of 3 mg/kg given on a once-weekly basis to tumor-bearing dogs were well-tolerated with no significant adverse events observed in this patient population and some potential hints of clinical anti-tumor activity (7). A distinct study evaluating a sorafenib dose of 5 mg/kg twice daily administered to dogs with un-respectable or diffuse hepatocellular carcinoma found that this schedule and dosage was tolerable; however, cutaneous toxicity (42.9%) and diarrhea (14.3%) occurred as adverse events. Nonetheless, none of these adverse events were considered dose-limiting (8).

Previous studies suggest that sorafenib has activity against canine tumor cell lines, including canine mammary tumor, hemangiosarcoma, osteosarcoma and urothelial carcinoma cell lines (9–12). Treatment of canine mammary tumor primary cell lines with sorafenib induced growth inhibition and prevented vascular mimicry structure formation *in vitro* (10). While these data provide support for the notion that sorafenib may exhibit anti-tumoral activity and inhibit angiogenesis in several canine tumor types, a more detailed understanding of the pharmacokinetic properties of sorafenib in dogs with spontaneous cancer is necessary to guide both dose and schedules for future clinical studies that more actively explore anti-cancer activity and pharmacodynamic effects. Given the lengthy half-life for this drug class and the potential for drug accumulation to occur in an older population of tumor-bearing dogs, the purpose of the following study was to assess the systemic exposures associated with oral doses of sorafenib at 3 mg/kg in pet dogs with a cancer diagnosis.

## Materials and Methods

### Eligibility

This clinical trial was conducted following Animal Care and Use Committee approval (Animal Clinical Investigation, Chevy Chase, MD). Written and informed consent was obtained from pet owners prior to clinical trial enrollment. Client owned dogs weighing ≥5 kg with a cytological and/or histologic diagnosis of cancer that had either failed standard therapy or for which the owners declined standard therapy were eligible for enrollment into this study. Eligible dogs included any breed, age, or sex deemed to be healthy with a favorable modified Eastern Cooperative Oncology Group (ECOG) performance status (0–1). Additional eligibility criteria included at least 21 days since prior chemotherapy (including systemic prednisone or non-steroidal anti-inflammatory drugs), radiotherapy, and/or surgery, and no evidence of other serious systemic disorder (e.g., cardiac or renal disease) considered incompatible with the study.

Initial screening included a baseline physical examination, complete blood count (CBC), chemistry panel, and urinalysis (UA). Patients with an absolute neutrophil count of <2.0 K/mL, platelet count of <100 K/mL, bilirubin >2 × upper limit of normal (ULN), and serum creatinine >1.4 were excluded. No criteria related to stage of disease or tumor burden were used to define eligibility in this tolerability and pharmacokinetic study.

### Drug Product

Sorafenib Tosylate bulk active pharmaceutical ingredient (API) was manufactured at a cGMP compliant facility and supplied by Tecoland Pharmaceuticals (Irvine, CA, USA). Sorafenib was compounded into oral capsules by Johnson Compound and Wellness (Waltham, MA, USA). The target dose of sorafenib was set at 3 mg/kg based on previous tolerability data from tumor-bearing dogs (7).

### Study Design

Six dogs were prospectively enrolled in this multicenter rolling-6 clinical trial evaluating the tolerability and pharmacokinetics of sorafenib in client-owned dogs with spontaneous malignancies (13). Dogs were enrolled at a dose cohort of 3 mg/kg given orally on a once-weekly basis. This dosing regimen was chosen based on prior studies demonstrating that sorafenib doses of 3 mg/kg were well-tolerated and associated with a suggestion of clinical activity in pet dogs with cancer (7). Each dog entered into the study was intended to receive at least one dose of sorafenib with the option to continue treatment if stable disease or an objective tumor response was observed at the request of the attending oncologist and principal investigator. All dogs received sorafenib capsules orally at 3 mg/kg in the morning with food. Dogs were fasted prior to sorafenib administration and blood samples (5 mL each) were collected prior to dosing and at 1, 2, 6, 12, 24, 48, 72, 96, and 168 h following drug administration. Based on the tolerability of the starting dose in 6 dogs, no additional dogs or dose cohorts were evaluated in this study. Clinical response to sorafenib therapy was not assessed in the study.

### Assessment of Adverse Events

Dogs were evaluated for adverse events (AEs) on days 1, 2, 3, 4, and 7 of study, as well as at interim time points as deemed necessary with physical exam and AE monitoring case reporting forms. AEs were defined as attributable to the drug, the disease, or study participation using the following modifiers: likely, probable, and unlikely. AEs attributed to disease included disease progression or signs and symptoms definitively related to disease. All AEs were defined and graded according to the published VCOG-CTCAE criteria (14).

### Blood Collection and Processing

Blood was collected into venous blood collection polyethylene terephthalate (PET) tubes containing no additives and samples were incubated at room temperature for 30 min, then centrifuged at 3,000 g × 15 min. Serum was transferred by pipette into fresh PET tubes and frozen at −20°C for <48 h. Frozen serum samples were shipped overnight on wet ice to a central location where samples were stored in −80°C until analysis. Serial blood collections were performed on all six dogs enrolled in this study; however, issues were encountered during sample transport that led to the loss of samples and inability to accurately analyze sorafenib serum concentrations from 2 study dogs.

### Bioanalysis of Sorafenib Serum Concentrations

Serum concentrations of sorafenib and its N-oxide metabolite were determined using ultra high-pressure liquid chromatography (UHPLC) with tandem mass spectrometry detection. Samples were analyzed on an UHPLC system consisting of an UltiMate 3000 Pump, Column Compartment and Autosampler (Thermo Fisher Scientific, Waltham, MA, USA) coupled to an Orbitrap mass spectrometer (Q Exactive Focus, Thermo Fisher Scientific). Briefly, 40 μL of either serum samples, calibration standards, serum quality controls (QCs), or serum blanks were added to 200 μL acetonitrile fortified with an internal standard, flunixin (20 ng/mL) to precipitate serum protein. Samples were vortexed for 5 s and centrifuged at 6,000 g × 10 min. 100 μL of supernatant was diluted with 100 μL water in an autosampler vial and samples centrifuged at 2,000 g prior to analysis.

Chromatographic separation was achieved on a Hypersil Gold Vanquish column (50 mm x 2.1 mm, 1.9 μm particle) with a gradient elution of 0.1% formic acid in water/0.1% formic acid in acetonitrile at a flow rate of 0.375 mL/min. Sorafenib and Sorafenib-N-oxide were eluted from the Hypersil Gold column at 3.20 +/−0.05 min and 2.79 +/−0.05 min after sample injection, respectively. Analyte detection was accomplished using parallel reaction monitoring in the positive electrospray ion mode with a spray voltage of 3.5 kV and a temperature of 300 C. A collision energy of 35 electron volts (eV) was used for fragmentation of all the analytes within the collision cell. For quantitation of each analyte species, fragment ions of 252.076, 270.87, and 406.056 m/z for sorafenib, 201.986, 229.061, and 286.082 m/z for sorafenib-N-oxide, and 239.061, 264.050, and 279.074 m/z for the internal standard, flunixin were used.

Quantitative results were obtained by batch processing raw MS data from sequences of serum blanks, calibration standards, QC's, and canine serum samples through a processing method that identified and integrated each peak in each sample and calculated the internal standard based calibration curve using a weighted (1/X) linear fit. Serum concentrations of sorafenib and sorafenib-N-oxide were calculated using Xcalibur software (Thermo Fisher Scientific). Sorafenib concentrations were determined by interpolation over the quantitation range of 2.0 ng/mL to 1,000 ng/mL. The limit of quantitation (LOQ) of sorafenib was 2.0 ng/mL with a limit of detection (LOD) of 0.5 ng/mL. The LOQ for sorafenib-N-oxide was 5.0 ng/mL with a LOD of 1.0 ng/mL.

## Results

### Patient Demographics

A total of six client-owned dogs (*n* = 5 castrated male; *n* = 1 spayed female) were enrolled in the rolling-six study from February 2020 – December 2020 (13). The median age was 10 years (range 4 – 15 years) and mean weight was 22.4 kg (range 12.2–28.8 kg). Breeds represented were Havanese (*n* = 1), Welsh Pembroke Corgi (*n* = 1), Golden Retriever (*n* = 1), and Mixed breed (*n* = 3). Tumor types included apocrine gland anal sac adenocarcinoma (*n* = 1), hepatocellular carcinoma (*n* = 1), histiocytic sarcoma (*n* = 1), oral malignant melanoma (*n* = 1), metastatic retroperitoneal hemangiosarcoma (*n* =1) and a metastatic carcinoma of unknown primary origin (*n* = 1) (Table 1). Three patients (50%) had received at least one previous chemotherapy protocol prior to enrolling in the trial. Three patients (50%) were naïve to chemotherapy prior to enrollment in the study.

**Table 1:** Patient demographics.

**Patient demographics**
**Total number of patients**	6
**Age (yr)**	
Median	10
Range	4–15
**Weight (kg)**	
Median	22.4
Range	12.2–28.8
**Gender (** ***n*** **)**	
Castrated male	5
Female spayed	1
**Tumor type (** ***n*** **)**	
Anal sac adenocarcinoma	1
Hepatocellular carcinoma	1
Histiocytic sarcoma	1
Oral malignant melanoma	1
Retroperitoneal hemangiosarcoma (metastatic)	1
Other carcinoma (metastatic, unknown primary origin)	1
**Prior therapy (** ***n*** **)**	
Yes	3
No	3

### Adverse Events and Analysis of Sorafenib Serum Concentrations

No patients in this study experienced AEs which were deemed attributable to sorafenib administration; however, dogs on this trial were not subject to chronic dosing. Therefore, the AE profile associated with chronic administration of sorafenib cannot be fully assessed in this patient population.

A primary objective of this study was to evaluate the systemic exposures associated with oral dosing of sorafenib administered at 3 mg/kg in cancer-bearing dogs. In prior human Phase I studies, the Cmax of sorafenib was determined to be approximately 6–12 h post-drug administration due to the relatively slow absorption phase. Additionally, prior assessment of relevant pharmacodynamic modulation in cancer cell lines demonstrated biochemical kinase selectivity (including inhibition of C-raf, wild-type B-raf, V599E mutant B-raf, VEGFR-2, c-KIT) for sorafenib at an IC_50_ ranging from 6 to 90 nM (15).

**Figure 1:**
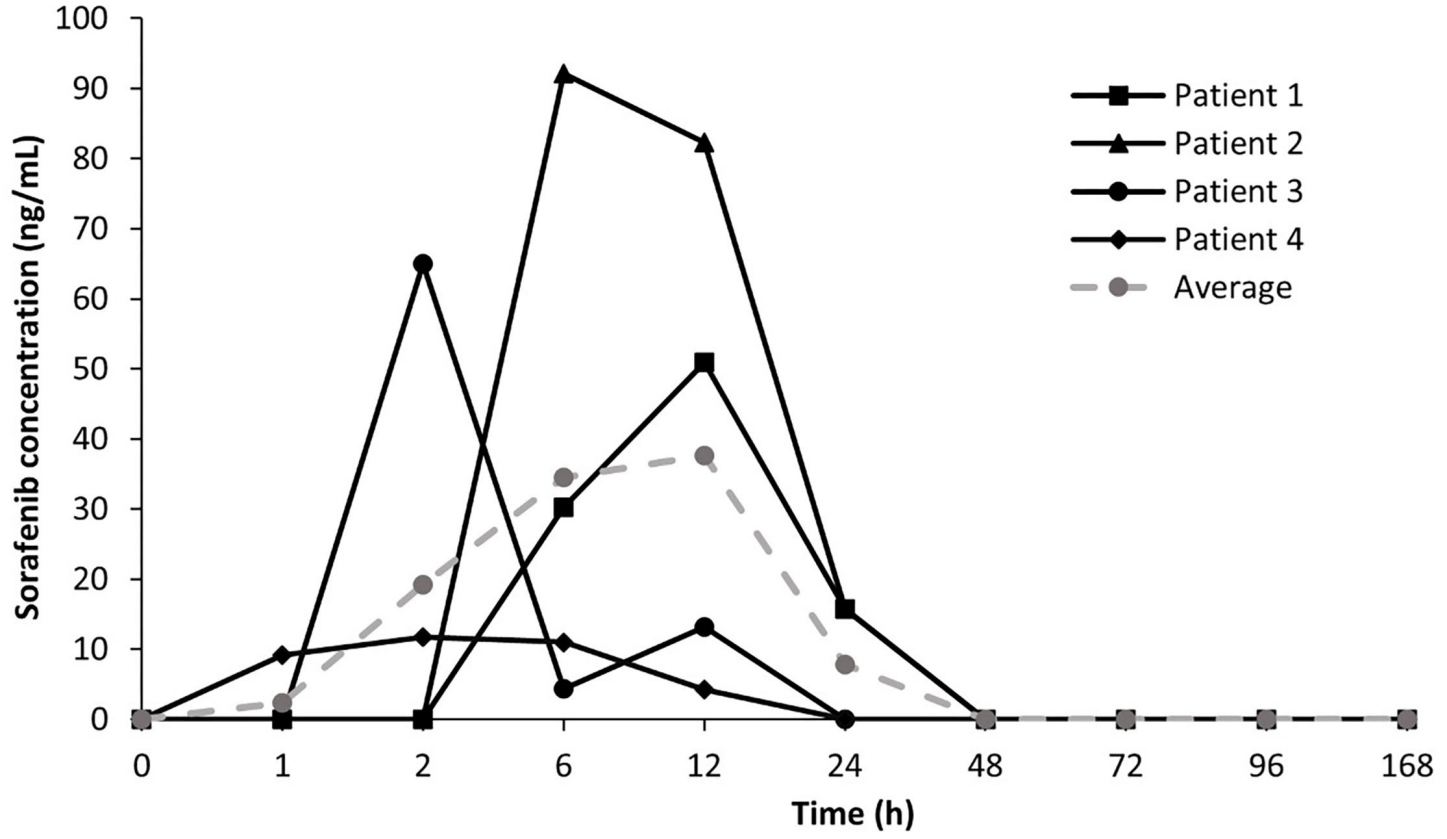
Time-course of sorafenib serum concentrations in tumor-bearing dogs. Mean and average sorafenib serum concentration – time profiles for four patients treated with oral sorafenib at 3 mg/kg.

Time-course of sorafenib serum concentrations for four tumor-bearing dogs orally administered sorafenib are shown in Figure 1. Administration of sorafenib at 3 mg/kg resulted in median time to peak serum concentrations at 4 h (range 2–12 h) (Supplementary Table 1). Mean sorafenib concentrations showed high interpatient pharmacokinetic variability which is concordant with clinical data demonstrating high variability in cumulative drug exposure in humans following sorafenib treatment (16). The Cmax of sorafenib after oral administration was established at 54.9 ± 33.5 ng/mL (118.2 ± 72.1 nM) in the study population which supports the notion that sorafenib doses of 3 mg/kg are adequate to achieve drug exposure associated with relevant target modulation *in vitro*. At the 24-h timepoint, mean sorafenib concentrations declined by over 70% relative to the peak concentrations consistent with the reported half-life of sorafenib (20 to 36 h) in humans. Collectively, these findings suggest the need for a twice daily dosing schedule of sorafenib in dogs to achieve exposures likely to consistently modulate cancer targets.

## Discussion

The purpose of this clinical trial was to evaluate the systemic exposure associated with oral doses of sorafenib at 3 mg/kg in pet dogs with cancer as a means to guide schedule selection in tumor-bearing dogs. The demographics of subjects within the current study population represent a diverse population with multiple tumor types in which a large proportion of patients (50%) had received prior chemotherapy, with an appropriate washout period. Similar to human subjects enrolled in Phase I clinical studies, veterinary patients enter early-stage clinical trials when there is no standard of care, the patient's cancer has become refractory to other treatments, a clinical trial is more financially feasible than standard of care, or the therapeutic upside of a novel agent is preferred over conventional therapies. Although laboratory dogs are commonly integrated into preclinical assessments of novel therapeutics, dogs with spontaneous tumors represent a heterogenous clinical population and thus cannot be directly compared to a population of purpose-bred research dogs that are receiving an investigational agent as part of a toxicology study (17). In our trial population of older, tumor-bearing dogs (afflicted with the systemic “syndrome” of cancer), there were no AEs which were deemed attributable to sorafenib administration; however, the primary endpoint of this study was to evaluate the PK exposures associated with a single dose of sorafenib. Thus, dogs on this trial were not subject to chronic dosing and the AE profile associated with chronic administration of sorafenib requires further evaluation.

Sorafenib drug concentrations measured in this exploratory study identified several pharmacokinetic features consistent with those previously reported in human Phase I clinical trials, including considerable interpatient PK variability and peak sorafenib concentrations occurring between 1 and 12 h (16). Sorafenib is a small lipophilic molecule with low-solubility and high permeability and following oral administration, it is rapidly absorbed from the gastrointestinal tract. In our study population, the time to peak drug concentrations (Tmax) were reached between 2- and 12-h following drug administration. Importantly, the average Cmax for dogs receiving oral sorafenib was established at 54.9 ng/mL (or 118.2 nM) in the study population which supports the notion that sorafenib doses of 3 mg/kg are adequate to achieve drug exposures within the range of putative biochemical target inhibition (15). High interpatient variability in systemic exposure was observed in dogs receiving oral sorafenib. This is concordant with reported findings from four Phase I monotherapy trials in human patients treated with sorafenib demonstrating considerable interpatient plasma concentration variability of sorafenib with no clear dose dependency (6). In addition, food intake has been reported to have no appreciable impact on sorafenib PK or metabolism. Interestingly, the observed interpatient PK variability does not appear to be related to the incidence or severity of drug-related AEs in humans receiving sorafenib (4). The small population size and low incidence of AEs in our canine study population preclude definitive statements regarding the severity of sorafenib-induced AEs and cumulative dose and sorafenib exposure level. Lastly, mean serum sorafenib levels declined by over 70% relative to peak concentrations by 24 h in all dogs. This decline in serum drug levels is consistent with the reported half-life of sorafenib (20 to 36 h) and suggests the need for a twice daily dosing schedule in dogs to achieve putative trough target concentration. Given the presumed long half-life of sorafenib in dogs, future tolerability studies in tumor-bearing dogs administered sorafenib on a twice daily dosing schedule are necessary to assess the potential for delayed adverse event development secondary to drug accumulation.

Given the exploratory nature of this study, several important limitations exist. The intent of the study was to determine the initial PK parameters associated with oral administration of sorafenib and therefore, drug levels are reflective of single drug dosing and do not capture steady state parameters which are typically achieved within 7 days of continuous dosing of sorafenib. Furthermore, the sampling interval in the current study was relatively limited and precluded determination of full PK parameters such as the apparent volume of distribution, terminal half-life and apparent drug clearance of sorafenib in dogs. The concurrent assessment of relevant pharmacodynamic modulation in study patient tumor tissues was beyond the scope of the current study; however, future pharmacodynamic studies in dogs receiving sorafenib would provide important confirmatory data that serum concentrations of sorafenib are associated with relevant therapeutic target (e.g., Raf kinases, VEGFR and PDGFR) modulation *in vivo*. Lastly, the number of dogs evaluated in this trial was small and patients were not subject to chronic sorafenib dosing; therefore, further assessment of the drug-related gastrointestinal and hematological adverse event profile associated with chronic administration of sorafenib in a larger population of tumor-bearing dogs is warranted. In summary, sorafenib administration at intended doses of 3 mg/kg in tumor-bearing dogs results in serum drug concentrations that may be associated with target kinase inhibition. The observed decline in serum sorafenib levels by 24 h suggests the need for a twice daily administration schedule for continuous putative target modulation. Future trials in dogs with spontaneous malignancies are recommended at this dosing schedule to determine clinical benefit and assess the effect of sorafenib administration on associated pharmacodynamic target modulation *in vivo*.

## Data Availability Statement

The original contributions presented in the study are included in the article/Supplementary Material, further inquiries can be directed to the corresponding author/s.

## Ethics Statement

The animal study was reviewed and approved by Animal Clinical Investigation, Chevy Chase, MD, USA. Written informed consent was obtained from the owners for the participation of their animals in this study.

## Author Contributions

CK, JC, SS, and SV designed the study methodology. CK, JC, and SS supervised the study. JM oversaw bioanalysis of sorafenib serum concentrations. JF, CK, JC, SS, and JM performed data analysis. JF wrote the original draft. CK, JC, SS, SV, and JM reviewed and edited the manuscript. All authors contributed to the article and approved the submitted version.

## Funding

This study was funded by Ethos Discovery (501c3).

## Conflict of Interest

The authors declare that the research was conducted in the absence of any commercial or financial relationships that could be construed as a potential conflict of interest.

## Publisher's Note

All claims expressed in this article are solely those of the authors and do not necessarily represent those of their affiliated organizations, or those of the publisher, the editors and the reviewers. Any product that may be evaluated in this article, or claim that may be made by its manufacturer, is not guaranteed or endorsed by the publisher.
