# Supplementary material for: Pharmacokinetic Exposures Associated With Oral Administration of Sorafenib in Dogs With Spontaneous Tumors
Source: Front Vet Sci. 2022 May 19;9:888483. doi: 10.3389/fvets.2022.888483 (PMC9162170; doi:10.3389/fvets.2022.888483)
Supplement: Supplementary file 1 [file Table_1.DOCX]

**Supplemental Table 1. Pharmacokinetic parameters**

|  |  |  |  |  |  |  |
| --- | --- | --- | --- | --- | --- | --- |
|  | **Sorafenib Concentration - ng/mL** | | | |  |  |
| **Hours** | **Patient 1** | **Patient 2** | **Patient 3** | **Patient 4** |  |  |
| 0h | 0.0 | 0.0 | 0.0 | 0.0 |  |  |
| 1h | 0.0 | 0.0 | 0.0 | 9.2 |  |  |
| 2h | 0.0 | 0.0 | *65.0 | *11.7 |  |  |
| 6h | 30.3 | *92.1 | 4.4 | 11.0 |  |  |
| 12h | *50.9 | 82.3 | 13.2 | 4.2 |  |  |
| 24h | 15.7 | 15.7 | 0.0 | 0.0 |  |  |
| 48h | 0.0 | 0.0 | 0.0 | 0.0 |  |  |
| 72h | 0.0 | 0.0 | 0.0 | 0.0 |  |  |
| 96h | 0.0 | 0.0 | 0.0 | 0.0 |  |  |
| 168h | 0.0 | 0.0 | 0.0 | 0.0 |  |  |
| *Designates time to maximum concentration (Tmax) | | | |  |  |  |
| Mean Cmax (ng/mL) | | 54.9 ± 33.5 |  |  |  |  |
| Mean Cmax (nM) | | 118.2 ± 72.1 |  |  |  |  |
